# A pilot study of peptide vaccines for VEGF receptor 1 and 2 in patients with recurrent/progressive high grade glioma

**DOI:** 10.18632/oncotarget.25131

**Published:** 2018-04-20

**Authors:** Shunsuke Shibao, Ryo Ueda, Katsuya Saito, Ryogo Kikuchi, Hideaki Nagashima, Atsuhiro Kojima, Hiroshi Kagami, Eriel Sandika Pareira, Hikaru Sasaki, Shinobu Noji, Yutaka Kawakami, Kazunari Yoshida, Masahiro Toda

**Affiliations:** ^1^ Department of Neurosurgery, Keio University School of Medicine, Shinjuku-ku, Tokyo 160-8582, Japan; ^2^ Department of Neurosurgery, Kawasaki Municipal Hospital, Kawasaki, Kawasaki-ku, Kawasaki, Kanagawa 210-0013, Japan; ^3^ Department of Neurosurgery, Ashikaga Red Cross Hospital, Ashikaga, Tochigi 326-0843, Japan; ^4^ Department of Neurosurgery, Saitama Municipal Hospital, Midori-ku, Saitama, Saitama 336-8522, Japan; ^5^ Department of Neurosurgery, Saiseikai Yokohamashi Tobu Hospital, Tsurumi-ku, Yokohama, Kanagawa 230-8765, Japan; ^6^ Division of Cellular Signaling, Institute for Advanced Medical Research, Keio University School of Medicine, Shinjuku-ku, Tokyo 160-8582, Japan

**Keywords:** high grade glioma, peptide vaccine, vascular endothelial growth factor receptor

## Abstract

**Object:**

Early-phase clinical studies of glioma vaccines have shown feasibility and encouraging preliminary clinical activity. A vaccine that targets tumor angiogenesis factors in glioma microenvironment has not been reported. Therefore, we performed a pilot study to evaluate the safety and immunogenicity of a novel vaccination targeting tumor angiogenesis with synthetic peptides for vascular endothelial growth factor (VEGF) receptor epitopes in patients with recurrent/progressive high grade gliomas.

**Methods:**

Eight patients received intranodal vaccinations weekly at a dose of 2mg/kg bodyweight 8 times. T-lymphocyte responses against VEGF receptor (VEGFR) epitopes were assessed by enzyme linked immunosorbent spot assays.

**Results:**

This treatment was well-tolerated in patients. The first four vaccines induced positive immune responses against at least one of the targeted VEGFR epitopes in the peripheral blood mononuclear cells in 87.5% of patients. The median overall survival time in all patients was 15.9 months. Two achieved progression-free status lasting at least 6 months. Two patients with recurrent GBM demonstrated stable disease. Plasma IL-8 level was negatively correlated with overall survival.

**Conclusion:**

These data demonstrate the safety and immunogenicity of VEGFR peptide vaccines targeting tumor vasculatures in high grade gliomas.

## INTRODUCTION

Despite the recent advances obtained by the introduction of chemoradiotherapy with temozolomide (TMZ), the overall outcome of patients with high grade gliomas, such as glioblastoma (GBM), anaplastic astrocytoma (AA), and anaplastic oligodendroglioma (AO), remains dismal [1]. Options are particularly limited for patients with recurrent disease, and novel treatments are needed.

Cancer immunotherapy has become an attractive therapeutic modality for cancer, and is regarded as the fourth modality of cancer treatment after surgery, radiotherapy and chemotherapy [2]. Early-phase studies of glioma peptide-based vaccines targeting tumor associated antigens that have the potential ability to induce tumor-specific T-cell responses have shown feasibility and encouraging preliminary clinical activity [3–13]. The ultimate success of glioma vaccines depends on the further refinement of strategies to target appropriate multiple glioma-associated antigens [14], and to reverse glioma-induced global immune-suppression through immune checkpoint blockade [15–17].

Anti-cancer therapeutic approaches that target the vascular endothelial growth factor (VEGF) or inhibit its receptors, VEGF-receptor (VEGFR) 1 and VEGFR2, have recently been developed [18–21]. VEGFR1 and VEGFR2 are induced in a tumor stage-dependent manner during glioma progression [22] and are exclusively expressed in tumor vascular endothelial cells [20, 23] and glioma cells [24, 25], suggesting that VEGF-receptors are promising targets for tumor endothelial cell specific therapy for glioma patients. Indeed, clinical studies of VEGFR peptide vaccines for patients with advanced gastrointestinal cancers and renal cell cancer exhibited feasibility and encouraging clinical activity [26, 27]. However, no clinical studies of a vaccine therapy targeting tumor angiogenesis factors such as VEGFR in high grade glioma patients has been reported. Thus, this study is the first to evaluate a peptide-based vaccine targeting tumor vasculatures in high grade gliomas. The HLA-A2402–restricted epitopes for this therapy included two that had been previously identified and evaluated for safety and potent immunogenicity in cancer patients: a VEGFR1-derived peptide (VEGFR1-1084) [28] and a VEGFR2-derived peptide (VEGFR2-169) [29, 30]. We hypothesized that this regimen would prove to be safe, and induce potent anti-glioma endothelial cell immune responses.

## RESULTS

### Demographics and clinical characteristics

A total of 9 patients, who were found to be HLA-A^*^2402 positive by DNA typing of HLA genomic variations, were enrolled in this study. Eight of the 9 patients who received 8 or more vaccinations (at least 2 months) were evaluated for further analysis. The remaining one patient dropped out from this study due to disease progression and was excluded from further analysis. Table 1 shows the characteristics of eight enrolled patients.

**Table 1:** Characteristics of 8 enrolled patients

Case No.	Age (yrs)	Sex	Dx	Op.	RT	Chemo/other agent	No. of progression episodes	corticosteroid dosing prior to vaccination	PS	IDH1	1p/19q codeletion
1	75	F	GBM	1	Con.(60Gy), SRS(36Gy)	TMZ, Bev.	2	none	2	WT	-
2	58	M	GBM	1	Con.(60Gy)	TMZ	1	1mg/day	1	WT	-
3	41	F	AA	1	Con.(50Gy), SRS(14.7Gy)	TMZ	2	1mg/day	2	R132H	-
4	62	F	GBM	1	IMRT(60Gy)	TMZ	1	none	1	WT	-
5	68	M	AO	4 (Biopsy 2)	Con.(50Gy)	TMZ	4	none	2	R132H	+
6	37	M	AA	3	Con., SRS	ACNU, PAV, TMZ	4	none	0	WT	-
7	59	M	GBM	2	Con.(60Gy)	TMZ, IFN	1	none	1	WT	-
8	62	M	GBM	1	Con.(60Gy)	TMZ	1	none	1	WT	-

Abbreviations: AA, anaplastic astrocytoma; ACNU, nimustine hydrochloride; AO, anaplastic oligodendroglioma; Bev., bevacizumab; Chemo, chemotherapy; Con., conventional; DA, diffuse astrocytoma; Dx, diagnosis; GBM, glioblastoma; IFN, β-interferon; IMRT, Intensity modulated radiation therapy; OA, oligoastrocytoma; Op., operation; PAV, procarbazine/ACNU/vincristine; PS, performance status; RT, radiotherapy; SRS, stereotactic radiosurgery; TMZ, temozolomide.

### Toxicity

No major toxicity was found in this study. During the vaccination therapy, all eight patients developed a reaction at the injection site and one patient developed an ulcer at the injection site (Grade 3). No delayed wound healing or gastrointestinal bleeding was found during the therapy. Other adverse events, such as arterial and venous thromboembolism, hypertension, and proteinuria, which were reported in the clinical study of bevacizumab, were not detected.

### CD8^+^ T-cell response

**Figure 1:**
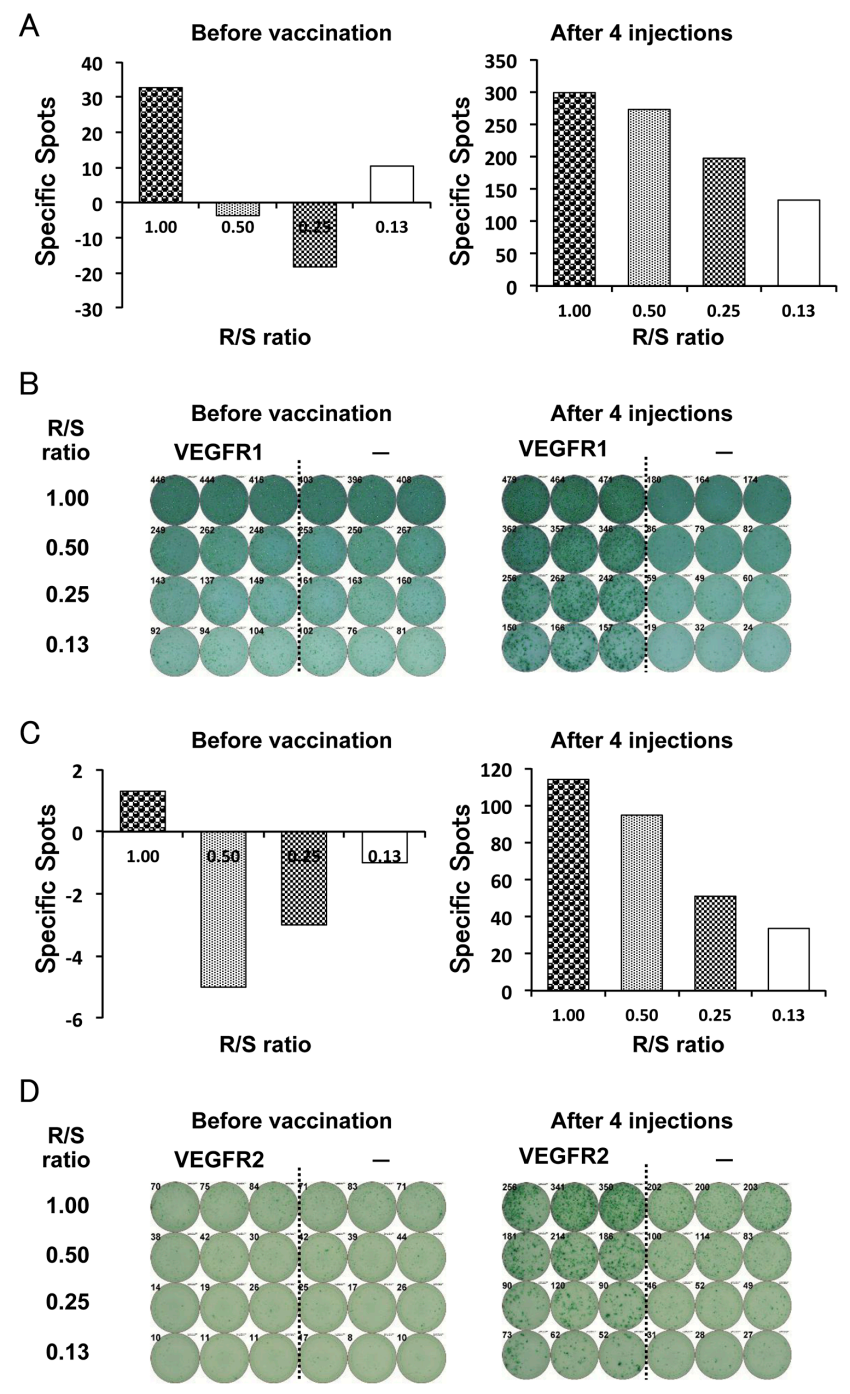
CD8^+^ T-cell response. Enzymelinked immunospot T (ELISPOT) assay was performed to examine immunological response using peripheral blood mononuclear cells (PBMCs) that were collected after every four injections (one month) A γ-interferon ELISPOT assay was performed against vascular endothelial growth factor receptor (VEGFR) 1 **(A and B)** and VEGFR2 **(C and D)** peptide or HIV peptide control (−) (Case 3). Average number of specific spots in each R/S ratio (A and C) and representative ELISPOT assay (B and D) of the same patient are shown. R / S, responder / stimulator ratio.

Seven (87.5%) of the eight patients who received more than eight injections (> 2 months) of the vaccination showed induced CD8^+^ T-cell response against VEGFR1-1084, and one patient (12.5%) showed induced CD8^+^ T-cell response against VEGFR2-169 (Table 2). One patient showed induced response to both VEGFR1 and VEGFR2 peptides, and one patient showed no response to the vaccination. The representative data from the γ-interferon enzyme-linked immunospot T (ELISPOT) assay and CTL responses in Case 3 before and after four injections (one month) are shown in Figure 1.

**Table 2:** Clinical results in 8 enrolled patients

Case No.	DTH	Toxicity	CD8^+^ T-cell before vaccination		CD8^+^ T-cell after vaccination		CD8^+^ T-cell positive response		Response	OS (days)
			VEGFR1	VEGFR2	VEGFR1	VEGFR2	VEGFR1	VEGFR2		
1	+	-	-	-	1+	-	+	-	PD	541
2	+	-	-	1+	3+	1+	+	-	SD	413
3	+	-	1+	-	3+	3+	+	+	PD	156
4	+	-	-	1+	2+	1+	+	-	PD	415
5	+	-	-	1+	3+	-	+	-	PD	673
6	+	-	-	1+	-	-	-	-	PD	965
7	+	-	1+	-	3+	-	+	-	SD	424
8	+	-	-	1+	3+	-	+	-	PD	530

Abbreviations: DTH, delayed-type hypersensitivity; OS, overall survival; PD, progressive disease; PR, partial response; SD, stable disease; VEGFR, vascular endothelial growth factor receptor; +, positive; -, negative.

### Clinical outcomes

Patients received a mean of 11.5 (±3.46) peptide vaccinations.

Two patients achieved stable disease (SD) and six patients (75%) revealed progressive disease (PD) at the end of eight vaccinations (Table 2).

**Figure 2:**
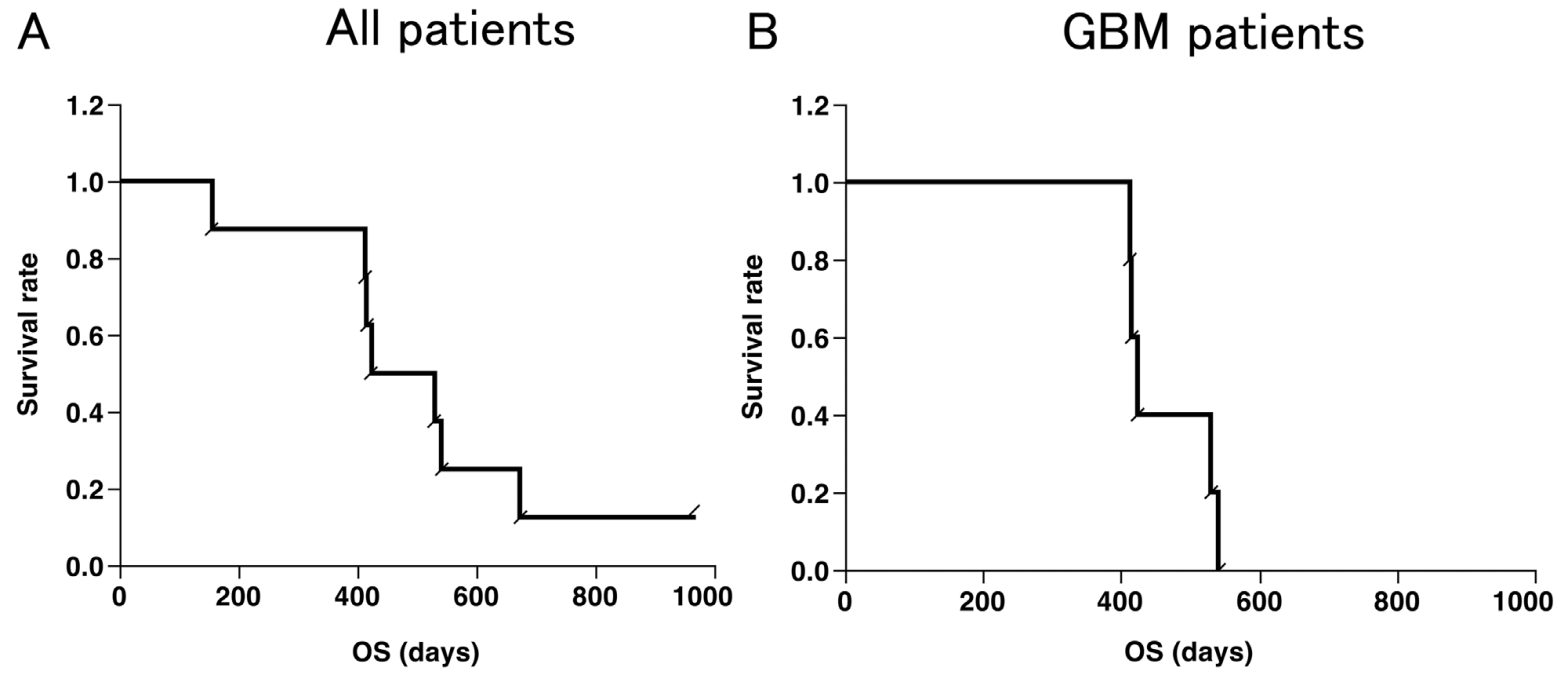
Kaplan-Meier curves Overall survival (OS) of all patients (n=8) **(A)** and GBM patients (n=5) (B), respectively. **(B)** The median OS was 447 days (15.9 months) and 1-year OS was 87.5 % in all patients. The median OS was 424 days (14.1 months) and 1-year OS was 100 % in GBM patients.

**Figure 3:**
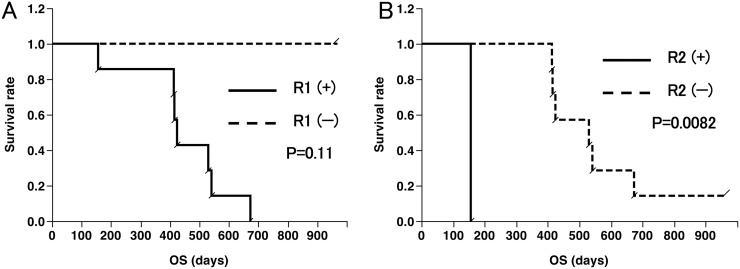
Overall survival (OS) curves according to CD8^+^ T-cell responses **(A)** OS of eight patients with seven patients with positive (+) and one patient with negative (−) response to VEGFR1 peptide; **(B)** OS of eight patients with one patient with positive (+) and seven patients with negative (−) response to VEGFR2 peptide.

At the time of analysis, one patient still had SD and was alive (2.7 years), and seven patients had died. The Kaplan–Meier curves for overall survival in all eight patients and five GBM patients are shown in Figure 2A and 2B, respectively. The median overall survival time (mOS) in all patients and GBM patients was 15.9 months and 14.1 months, respectively. One-year OS was 87.5 % for all patients and 100% for GBM patients. There was no significant correlation between the overall survival and increase in VEGFR-specific CD8^+^ T-cell frequencies in the Peripheral blood mononuclear cells (PBMCs) of patients after vaccination (Figure 3).

**Figure 4:**
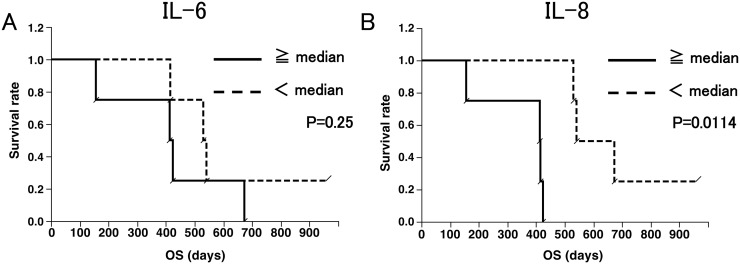
Overall survival (OS) curves according to IL-6 **(A)** and IL-8 **(B)** levels. (A) OS of eight patients, with four patients with IL-6 more than median (0.505 pg/ml) and four patients with IL-6 less than median. (B) OS of eight patients, with four patients with IL-8 more than median (2.81 pg/ml) and four patients with IL-8 less than median. IL-8 levels correlated with better survival. (p = 0.0114; Wilcoxon test).

We classified patients into two groups according to TNF-Ɑ, IL-6 and IL-8 levels after four vaccinations. Although TNF-Ɑ and IL-6 were not significantly related to OS, the patients with IL-8 levels lower than median (2.81 pg/ml) showed significantly longer OS than those with IL-8 levels higher than median (p=0.0114) (Figure 4).

## DISCUSSION

To our knowledge, this is the first clinical evaluation of peptide-based vaccines targeting tumor angiogenesis factors in high grade glioma patients. Our findings demonstrated the safety and immunogenicity, as well as preliminary efficacy of this approach.

Clinical trials of peptide-based vaccine therapy using the same VEGFR-derived/ HLA-A2402–restricted epitopes have been conducted to assess safety, tolerability, and potential clinical activity in patients with advanced gastrointestinal cancers and renal cell cancer [26, 27]. In the present study, we treated high grade glioma patients with a dose of VEGFR peptide that was previously determined to be safe. Furthermore, adverse effects to vaccination were limited to local erythema at the injection sites of the vaccine, indicating the safety of the VEGFR peptide vaccines. Adverse events, such as bleeding, wound-healing complications, arterial and venous thromboembolism, hypertension, and proteinuria, which were reported in the clinical study of bevacizumab, were not detected in this study.

This study is the first to document *in vivo* induction of specific CD8^+^ T-cell responses against two epitopes derived from tumor angiogenesis factors, VEGFR1-1084 and VEGFR2-169, in high grade glioma patients. Positive immune responses against VEGFR1 and VEGFR2 in PBMCs were induced in 87.5% and 12.5% of patients, respectively, suggesting that the VEGFR1-derived epitope was immunogenic in advanced high grade glioma patients. We did not find a correlation between the overall survival and the increase in the frequency of VEGFR-specific CD8^+^ T-cells among the PBMCs of patients after vaccination. While VEGFR2 is frequently expressed in the vasculature (100%) and tumors (33%) of the patients with GBM, VEGFR1 is expressed in the vasculature and tumors of only 20% of the patients with GBM [20]. The CTL response of the PBMCs of the patients prior to vaccination was 25% for VEGFR1 and 62.5% for VEGFR2 (Table 2). This CTL response might contribute to the appreciable OS. The same phenomenon was observed in the clinical study of Wilms tumor 1 peptide vaccination. Izumoto et al. reported that, after the vaccination, CTLs in the responders might change qualitatively, but not quantitatively [9]. Similarly, previous cancer immunotherapy trials have shown a poor correlation between clinical response and increase in antigen-specific CD8^+^ T-cell frequencies [9, 31–34]. With regard to the lower immunogenicity of the VEGF2-derived epitope than that derived from VEGFR1, further studies are warranted to determine whether the VEGFR2-specific immune response can be induced more effectively in newly-diagnosed patients with glioma, with higher immunoresponsiveness, than immunosuppressed patients with recurrent high-grade glioma.

Based on our data showing a negative correlation between plasma IL-8 levels and overall survival, further studies are warranted to determine whether production of IL-8, which promotes progression of numerous malignancies, including high grade gliomas [35, 36], can be a surrogate measure of vaccine efficacy in future trials. Moreover, evaluation of other immunological biomarkers may lead to better understanding of the critical immune response indicators that may help to predict clinical responses.

Recently, immunotherapy response assessment in neuro-oncology (iRANO) was reported to assess the clinical response of immunotherapy including vaccine therapy [37]. In iRANO, progressive disease is not confirmed for 6 months unless significant clinical decline unrelated to a comorbid event or concurrent medication is identified. In fact, one patient showed long SD in spite of PD at the end of eight injections (two months). Since progression confirmation is difficult in this protocol, we assessed the overall survival of patients to evaluate potential clinical activity of this therapy. In our study, the median overall survival from the initial vaccination in all patients and GBM patients was 15.9 months and 14.1 months, respectively. These results are comparable to those reported in the literature for previous clinical studies of glioma vaccines [5, 9, 38] and various combination regimens of chemotherapy and/or radiotherapy for recurrent GBM patients, although the sample size was relatively small.

Agents targeting the VEGF/VEGF receptor axis in GBM have widely been tested [39]. However, recent phase III trials in newly-diagnosed GBM patients demonstrated a failure of the monoclonal anti-VEGF-directed antibody bevacizumab to extend overall survival when combined with chemotherapy and radiation therapy, despite benefits in progression-free survival and quality of life [40, 41]. The studies in which bevacizumab was administered as monotherapy showed a median OS of 6.5-10.5 months [42–45], whereas our study showed median OS of 14.1 months in GBM. We cannot directly compare the results of the bevacizumab studies from those of our study due to many factors, such as variety of previous treatment, various histology, and different eligibility criteria. However, our VEGFR peptide-based vaccine may offer several advantages compared to bevacizumab therapy. First, although bevacizumab blocks only VEGF-A, however other members of the VEGF family, including VEGF-B to -E, help tumor angiogenesis. Our vaccine therapy against VEGFR1 and VEGFR2 may block the effects of VEGF-A to –E [46]. Second, VEGFR-specific CD8^+^ T cells induced by our peptide-based vaccine may be able to kill not only VEGFR-expressing glioma endothelial cells but also glioma cells, based on the evidence for the existence of tumoral VEGFR1/VEGFR2 expression [24, 25, 47]. Taken together, the combined application of VEGFR peptide-based vaccine and bevacizumab therapy may exhibit additive effects in clinical activity for high grade glioma patients.

This study has some limitations. Firstly, our study population was heterogenous, including both glioblastoma and other high-grade gliomas. Secondly, since the number of patients was small, the survival curves are only meant for reference. Thirdly, we included some confounding factors, such as molecular characteristics, time from diagnosis to treatment, and tumor mass.

In order to assess the safety, feasibility, and immunogenicity of the VEGFR peptide-based vaccine targeting tumor angiogenesis, we performed a pilot study for HLA-A2402 patients with recurrent/progressive high grade gliomas. The safety and the immunogenicity of this vaccine therapy have been verified, and the data suggest that VEGFR1/VEGFR2 vaccination may improve overall survival in this small population. However, definitive evidence of efficacy will require a future study of combination therapies with these vaccines, and standard treatment for newly-diagnosed high grade gliomas as an adjuvant setting, which is currently in the planning stages, as well as the immune-checkpoint blockade therapies.

## MATERIALS AND METHODS

The study protocol was approved by the institutional ethics committee.

### Patient eligibility

Patients diagnosed with high grade glioma including glioblastoma were enrolled in this study from May 2012 to August 2013 at Keio University Hospital (Tokyo, Japan).

Inclusion criteria were: 1) histological diagnosis of high grade glioma (WHO grade III or IV), 2) patients who had PD at study entry, 3) HLA genotyping; HLA-A^*^2402, 4) age between 16 and 80 years, 5) Eastern Cooperative Oncology (ECOG) performance status (PS) 0-2, 6) completion of standard treatment [surgical therapy (ST) + radiation therapy (RT) + temozolomide], 7) four-week interval from last chemotherapy or radiotherapy, 8) adequate bone-marrow, cardiac, pulmonary, hepatic and renal functions including neutrophil ≥1,000/μl, platelet count ≥50,000/μl, hemoglobin ≥8g/dL, plasma aspartate aminotransferase and alanine aminotransferase levels ≤4 times the normal limit, plasma bilirubin levels ≤1.5 times the normal limit, plasma albumin levels ≥ 2.5 g/dL, plasma creatinine levels ≤ 2.0 mg/dL, 9) life expectancy > 3 months, 10) signature confirming informed consent.

Exclusion criteria were: 1) uncontrollable infection, 2) the presence of another serious disease such as uncontrolled diabetes, hepatic disorder, cardiac disease, hemorrhage/bleeding, 3) total parenteral nutrition, 4) multiple cancers, 5) Myelodysplastic syndrome (MDS), MDS/myeloproliferative disease (MPD), and MPD, 6) allogenic hematopoietic stem cell transplantation, 7) severe immunological disorders (autoimmune disease, immunosuppression), 8) anaphylaxis to synthetic peptides 9) concurrent treatment with steroids or immunosuppressive agents, 10) pregnant or breast-feeding women, 11) mental disorder, 12) unhealed wound, 13) decision of unsuitability by the principal investigator or the physician in charge.

### Design of the VEGFRI/II vaccine therapy

This study was a non-randomized, open label clinical trial with VEGFR1-1084 and VEGFR2-169 vaccines for recurrent/progressive high grade glioma.

The primary end-points of this study were the safety of the peptide vaccination and the median OS time. The secondary end-points were immunological responses.

### Radiologic response monitoring and other clinical end points

Tumor size was assessed at weeks 9, 17, 25, and 33, and every 3months thereafter using magnetic resonance imaging (MRI) scans with contrast enhancement. Response was evaluated by the Response Evaluation Criteria in Solid Tumors (RECIST) by gadolinium (Gd) -enhanced T1 weighted images on the basis of the appearance of the pretreatment MRI [48]. OS was defined by the interval from study entry to date of death. MRI scans were used to evaluate time to progression.

### Treatment protocol

The dose of the peptide was determined to be 2mg/kg body weight based on a previous study [49]. Safety was verified by the study conducted for patients with advanced pancreatic cancer [49].

VEGFR1 (VEGFR1-A24-1084) and VEGFR2 (VEGFR2-A24-169) were emulsified in incomplete Freund’s adjuvant (Montanide ISA-51VG; SEPPIC, Paris, France) and administered subcutaneously close to an axillary or inguinal lymph node 8 times weekly. Patients treated more than 8 times (> 2 months) were enrolled. Administration of peptide vaccines was continued even after 8 injections if the patients consented.

### Toxicity assessment

Toxicity was assessed based on the common terminology criteria for adverse effects version 4.0 (CTCAE ver. 4.0). Toxicity was defined as a toxicity of grade 4 or greater.

### Peptides

GMP-grade VEGF1-1084 (SYGVLLWEI) and VEGFR2-169 peptides (RFVPDGNRI) were synthesized by the American Peptide Company (Sunnyvale, CA, USA) according to a standard solid-phase synthesis method, and purified by reversed-phase high-performance liquid chromatography (HPLC). The purity (>90%) and the identity of the peptides were determined by analytical HPLC and mass spectrometry analysis, respectively. The VEGFR peptides were provided by the Human Genome Center, The Institute of Medical Science, the University of Tokyo (Tokyo, Japan). The VEGF1-1084 and VEGFR2-169 peptides, and the epitope peptides derived from the HIV–Envelope protein restricted with HLA-A^*^2402 (RYLRDQQLL), were used for the measurement of CD8^+^ T-cell responses.

### CD8^+^ T-cell responses to peptide stimulation

To evaluate the specific CD8^+^ T-cell response, an ELISPOT assay was performed following *in vitro* expansion. PBMCs were obtained from patients before the vaccination treatment and after every four injections (1 month), and then frozen and stored in liquid nitrogen until use. Frozen PBMCs derived from the same patient were thawed concurrently, and their viability was confirmed to be >90%. PBMCs (5 × 10^5^/ml) were cultured with 10 μg/ml of the respective peptide and 100 IU/ml of interleukin-2 (Novartis, Emeryville, CA) at 37°C with 5% CO_2_ for 2 weeks. The peptide was added to the culture at Day 0 and 7 (final concentration 10 μg/ml). After incubation and CD4 depletion, harvested cells were used as responder cells in the ELISPOT assay, and peptide-pulsed TISI cells were used as stimulator cells (1 × 10^5^ cells per well). Non-peptide-pulsed TISI cells were used as negative control stimulator cells. Prepared harvested cells were cultured with peptide-pulsed TISI cells (2 × 10^4^ cells/well) at 1/1, 1/2, 1/4, and 1/8 ratio of responder cells and stimulator cells (R/S ratio) in a 96-well plate at 37°C overnight. Spots were captured and analyzed using an automated ELISPOT reader, ImmunoSPOT S4 or S5 (Cellular Technology Ltd, Cleveland, OH, USA) and ImmunoSpot Professional Software version 5.0.3 (Cellular Technology Ltd). The positivity of CD8^+^ T-cell responses was identified when the average spot-forming cells per well in response to the respective peptide was ≥10 spot-forming cells per well in response to the control peptide. The degree of positivity was assessed as the follows: 1+: 10–199, 2+: 200–299, 3+: 300–. Positive response of the CD8^+^ T-cells due to vaccination was defined as positivity increase after vaccination.

### Delayed-type hypersensitivity reaction

To test the cell-mediated cytotoxicity response, we observed skin reaction at the injected site. A positive delayed-type hypersensitivity (DTH) skin test reaction was defined as greater than grade 1 induration based on CTCAE (ver. 4.0) after vaccination.

### Cytokine measurements

Concentrations of TNF-Ɑ, IL-6 and IL-8 were measured in 25 μL of EDTA plasma using an ultrasensitive electrochemiluminescence immunoassay, according to the manufacturer’s instructions (Meso Scale Discovery: MSD; Rockville, MD). Ultrasensitive 10-plex plates were custom-designed for the Meso Scale Discovery 6000 instrument. Controls for standard curves were included in each plate. Samples with cytokine values less than the limit of detection were assigned a value of one half the limit of detection.

### Molecular-genetic analysis

Chromosomal number aberrations (CNAs) were assessed by metaphase comparative genomic hybridization, as described previously [50, 51]. Briefly, crude tumor DNA from formalin-fixed paraffin-embedded (FFPE) tissue was amplified by degenerate oligonucleotide primed-polymerase chain reaction (DOP-PCR), and labeled with another DOP-PCR, using digoxigenin (DIG)-11-dUTP (Roche, Mannheim, Germany). The reference DNA was amplified from 50 ng of DNA from a healthy man or woman, and labeled with biotin-dUTP (Roche). The probe mixture was denatured and hybridized to normal metaphase spreads (Vysis, Downers Grove, IL, USA). Unhybridized probes were washed out, and the metaphase spread was incubated with a fluorescein isothiocyanate-conjugated anti-DIG antibody (Roche) and rhodamine-conjugated avidin (Roche). Preparations were washed and counterstained with 4,6-diamino-2-phenylinodole in antifade solution. Red, green, and blue images were acquired, and ratios of fluorescence intensity along chromosomes were quantitated using the CytoVision® Analysis System (Applied Imaging, San Jose, CA, USA).

Mutation of the *IDH1* gene genes was assessed as follows: FFPE tissue sections were examined for *IDH1* R132H using immunohistochemistry, with an anti-mutant IDH1 antibody (Dianova, Hamburg, Germany) [52].

### Statistical analysis

All statistical analyses were performed with JMP 8.0.2 (SAS Institute, Cary, NC).

Statistical analyses were performed using the log-rank test. OS curves were estimated using Kaplan–Meier methodology. Differences were considered to be statistically significant when p < 0.05.

## Abbreviations

AA: anaplastic astrocytoma
AO: anaplastic oligodendroglioma
CNA: chromosomal number aberration
CTCAE: common terminology criteria for adverse effects
DIG: digoxigenin
DOP-PCR: degenerate oligonucleotide primed-polymerase chain reaction
DTH: delayed-type hypersensitivity
ECOG: Eastern Cooperative Oncology
ELISPOT: enzyme-linked immunospot T
FFPE: formalin-fixed paraffin-embedded
GBM: glioblastoma
Gd: gadolinium
HPLC: high-performance liquid chromatography
iRANO: immunotherapy response assessment in neuro-oncology
mOS: median overall survival
MDS: myelodysplastic syndrome
MPD: myeloproliferative disease
MRI: magnetic resonance imaging
OS: overall survival
PBMC: peripheral blood mononuclear cell
PD: progressive disease
PS: performance status
RECIST: Response Evaluation Criteria in Solid Tumors
RT: radiation therapy
SD: stable disease
ST: surgical therapy
TMZ: temozolomide
VEGF: vascular endothelial growth factor
VEGFR: vascular endothelial growth factor receptor.
